# Preparation, Physicochemical Characterization and Anti-Fungal Evaluation of Amphotericin B-Loaded PLGA-PEG-Galactosamine Nanoparticles

**DOI:** 10.34172/apb.2021.044

**Published:** 2020-07-15

**Authors:** Ghobad Mohammadi, Mostafa Fathian-Kolahkaj, Pardis Mohammadi, Khosro Adibkia, Ali Fattahi

**Affiliations:** ^1^Pharmaceutical Sciences Research Center Health Institute, Kermanshah University of Medical Sciences, Kermanshah, Iran.; ^2^Student Research Committee, Kermanshah University of Medical Sciences, Kermanshah, Iran.; ^3^Faculty of Pharmacy, Tabriz University of Medical Sciences, Tabriz, Iran.

**Keywords:** Amphotericin B, *Candida albicans*, Galactosamine, PLGA, PEG, Nanoparticles

## Abstract

***Purpose:*** The present study aimed to formulate PLGA and PLGA-PEG-galactosamine nanoparticles (NPs) loaded with amphotericin B with appropriate physicochemical properties and antifungal activity. PLGA was functionalized with GalN to increase the adhesion and antifungal activity of NPs against *Candida albicans*.

***Methods:*** The physicochemical properties of NPs were characterized by particle size determination, zeta potential, drug crystallinity, loading efficiency, dissolution studies, differential scanning calorimeter (DSC), X-ray powder diffraction (XRPD), and Fourier transform infrared (FT-IR). Antifungal activity of the NPs at different drug/polymer ratios was examined by determining minimum inhibitory concentrations (MICs).

***Results:*** the FT-IR and ^1^ HNMR analysis successfully confirmed the formation of PLGA- PEG-GalN NPs. The *PLGA* NPs were in the size range of 174.1 ± 3.49 to 238.2±7.59 nm while PLGA-GalN NPs were 255.6 ±4.08 nm in size , respectively. Loading efficiency was in the range of 67%±2.4 to 77%±1.6, and entrapment efficiency in the range of 68.185%±1.9 to 73.05%±0.6. Zeta potential and loading efficiency for PLGA-GalN NPs were –0.456, 71%. The NPs indicated an amorphous status according to XRPD patterns and DSC thermograms. The PLGA-PEG-GalN NPs showed higher fungistatic activity than PLGA NPs.

***Conclusion:*** the results demonstrated that the antifungal activity of PLGA-PEG-GalN NPs was higher than pure amphotericin B and PLGA NPs.

## Introduction

Amphotericin B, despite its toxicity, is the most reliable polyene antifungal drug to treat the severe systemic fungal infections including candidiasis, aspergillosis, coccidioidomycosis,blastomycosis, histoplasmosis, and idomycosis.^1-3^ Therefore, considerable effort is necessary for the development of different formulations.

Over the past several decades, nanoparticles (NPs) have received much more attention in a wide range of biomedical and technological applications, especially drug delivery.^4,5^ Polymeric nanoparticle-based therapeutics due to reduced side effects besides more flexibility, increased residence time in the body, and sustained and tunable release have been emerged as particulate carriers for targeted and controlled drug delivery.^6,7^ Among the different polymers developed to formulate polymeric NPs, poly (lactic-co-glycolic acid) (PLGA) is one of the most attractive polymeric candidates for drug delivery.^8,9^ PLGA is comprised of DL-lactic acid (LA) and glycolic acid (GA) monomers.^10^ The ester linkage of PLGA goes hydrolysis in aqueous systems, releasing the original monomers which are easily metabolized in the body.^11,12^

Nano-carriers can be grafted with specific targeting ligands such as sugar and vitamins leading to increase the specificity of delivered drugs, drug uptake and decrease their side effects compared to traditional dosage forms.^13-15^ Important amino sugars such as glucosamine and galactosamine are used for biological and biomedical purposes.^16-18^

The aim of the present research was the chemical conjugation of GalN to PLGA to increase the adhesion of drug nanoparticle to fungal cells. The PLGA- PEG-GalN NPs and PLGA NPs with different drug polymer ratios were assessed for the physicochemical properties and antifungal activity. Also, the minimum inhibitory concentrations (MICs) were investigated along with the kinetic drug release pattern of NPs.

## Materials and Methods

### Materials

Amphotericin B, galactosamine, polyethylene glycol (PEG), 4-dimethylaminopyridine (DMAP), ethylene dichloride (EDC), dimethyl sulfoxide (DMSO), dichloromethane (DCM), acetone, succinic anhydride, and acetonitril were obtained from Merck (Germany). PLGA (50:50 D, L-lactide:glycolide) and polyvinyl alcohol (PVA) were obtained from Purac (Gorinchem, the Netherland) and Acros (Acros Organics, Geel, Belgium), respectively.

### Synthesis of PLGA-PEG-GalN copolymers

PLGA-PEG-Gal N synthesize in three steps.^19,20^ At first, polyethylene glycol diacid (HOOC–PEG–COOH) was prepared by the reaction of carboxyl group of succinic anhydride with alcohol group of PEG in order to from the ester band. For this purpose, 2 g (0.33 mmol) polyethylene glycol (PEG), 46.6 mg (0.46 mmol) Succinic anhydride, and 48.8 mg (0.399 mmol) DMAP were dissolved in 10 mL DCM and stirred at 0°C for 2 hours. This mixture was again stirred under nitrogen gas at room temperature overnight. The resulting solution was precipitated in cold diethyl ether and washed by water. This precipitate dissolved in DCM and then re-precipitated by cold diethyl ether.

At second step, GalN was conjugated to the previous product by the amidation reaction as follows: 50 mg of the previous product (0.13 mmol), 50 mg (0.26 mmol) EDC, and 30.2 mg (0.26 mmol) NHS were dissolved in 20 mL of DCM. The resulting mixture was then stirred under hydrogen gas at room temperature for 6 hours. 36.44 mg (0.169 mmol) of GalN was dissolved fully in dry DMSO and injected into the mixture. The solution was then mildly stirred for 72 hours and then dialyzed using a membrane with a molecular weight cut off of 3.5 kDa for 12 hours.

Finally, PLGA was conjugated to PEG-GalN by the esterification reaction as follows: 50 mL (0.833 mmol) of the previous product and PLGA (183.3 mL, 0.461 mmol) were dissolved in DCM (20 mL) at 0°C. 0.01 mmol DMAP was added to mixture and resulting solution was precipitated into diethyl ether. The precipitate was re-dissolved in DCM and re-precipitated in cold diethyl ether for more purification. The solution was recovered by filtration and washed repeatedly with water to remove unreacted PEG-GlaNs.

### ^1^H-nuclear magnetic resonance spectroscopy (^1^H-NMR)

The ^1^H-NMR analysis of samples was performed at 400 MHz on a Varian Unity 400 spectrometer. All samples were dissolved in either deuterated DMSO or D_2_O.

### Fourier transforms infrared (FT-IR) spectroscopy 

The FT-IR spectra of the samples were recorded by the KBr disc technique with an IR Prestige-21 (Shimadzo. Co, Japan). The scanning range was 450-4000 cm^-1^ at a resolution of 4 cm^-1^.

### Preparation of PLGA NPs

Briefly NPs with 1:1 1:3 and 1:6 ratios of the drug to PLGA were prepared using quasi emulsion solvent diffusion technique.^21^ Amphotericin B and PLGA were co-dissolved in 3 mL DMSO. Organic phases was slowly (2 mL/min) poured into the 40 mL of aqueous solution containing PVA (1% w/v) as an emulsifier. The resultant mixture was homogenized at 13 000 rpm for 5 min in an ice-water bath during processing. The NPs were precipitated by ultra-centrifuge in order to remove free drug and impurities of NPs. The precipitates were collected and washed twice by distilled water and freeze-dried for 24 hours to produce dry powders.

### PLGA-PEG-GalN NPs

The procedures for PLGA-PEG-GalN NPs preparation were the same as described above for PLGA NPs; except the PVA concentration that was kept at 1% w/w in the aqueous phase because of the more hydrophilic properties of PLGA.

### Nanoparticle size and zeta potential 

The particle size, zeta potential, and polydispersity index of NPs were assisted by dispersing the precipitated NPs in 1 mL DI water. These characteristics were measured via a Zetasizer (Nano-ZS Malvern Instrument Ltd., Worchestershine, UK). All the measurements were observed with visible red laser wavelength at 632.8 nm. The experiments have been repeated three times.

### Determination of process yield and loading efficiency 

Each samples (20 mL) was dissolved in 50 mL of DMSO and then sonicated (Tecno3, Tecno-Gaz, Italy) for 5 minutes.^21^ The concentration NPs solutions was measured using direct UV absorbance detection at 371 nm. Loading efficiency (LE) and process yield were calculated according to the follow^22^:

(1) $$\text{Loading Efficiency }\left(\text{LE\%}\right)=\;\frac{\text{Weight of drug entrapped in NPs}}{\text{Initial weight of drug added}}\times 100$$

(2) $$\text{Process yield }\left(\text{PY \%}\right)=\;\frac{\text{NPs weight }}{\text{total amount of solids}}\times 100\;\left(2\right)$$

### SEM analysis 

The morphology of NPs was observed by using scanning electron microscopy (SEM) (Leo Electron Microscopy Ltd., Cambridge, UK). Samples were placed on an aluminum stub and coated with gold in an argon atmosphere.

### DSC analysis 

Thermal behavior of samples were studied with a differential scanning calorimeter (DSC) (Shimadzu, Japan) from 20 to 260°C at a heating rate of 40°C/min. For DSC Procedure, approximately 3 mg of samples were placed on the aluminum pans and sealed.

### X*-*ray powder diffractometry (XRPD)

The XRPD data were collected on the X-Ray diffractometer (Siemens, Model D5000, and Germany) and the target was Cu Kα with a wave length of 1.54060 A°. The measurements were performed at 40 kV and 20 mA. Diffractograms were scanned over a 2θ range of 5–60° with scanning rate of 0.06°/min.

### In vitro drug release and release kinetic study

The release rate of amphotericin B from the NPs was determined by dialysis method and quantified by high performance liquid chromatography.^23^ One milligram amphotericin B equivalent of various formulations were placed in a flask containing phosphate buffer saline with 5% v/v of DMSO (200 mL) and were incubated at 37°C in an incubator shaker with speed of 100 rpm. After 30 minutes, samples (1 mL) were collected at specific intervals (0.5, 1, 2, 4, 8, 10, 12, 22, 24, 48, 72 and 96 hours) and replaced with PBS:DMSO (95:5 % v/v) while maintaining strict sink condition throughout the experiment.

The kinetics of drug release was fitted into 11 types of mathematical models including zero order, first order, Peppas, Peppas-Sahlin, Hixson-Crowell cube root, Higuchi square root, Square root of mass, three seconds root of mass, logarithmic probability, linear probability, and Weibull model to find out mechanism of drug release. The best prediction ability of the kinetic models was determined by calculation of squared correlation coefficients and minimum prediction error.

## Results and Discussion

### Conjugation of PLGA and GalN

**Figure 1:**
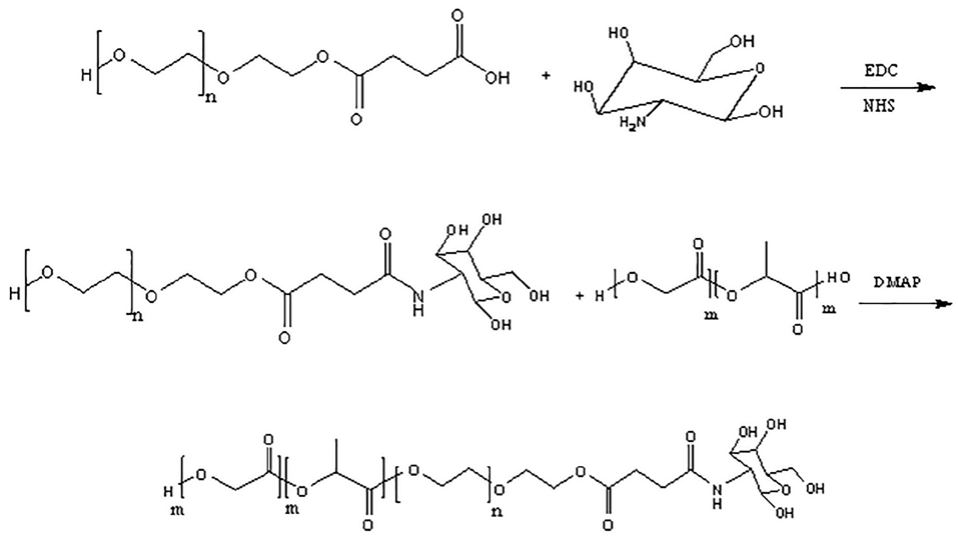
FT-IR of the PLGA (a), PEG (b), GalN (c), PEG-Succinic anhydride (d), PEG-Succinic anhydride-GalN (e), and PEG-Succinic anhydride- GalN-PLGA (f).

**Figure 2:**
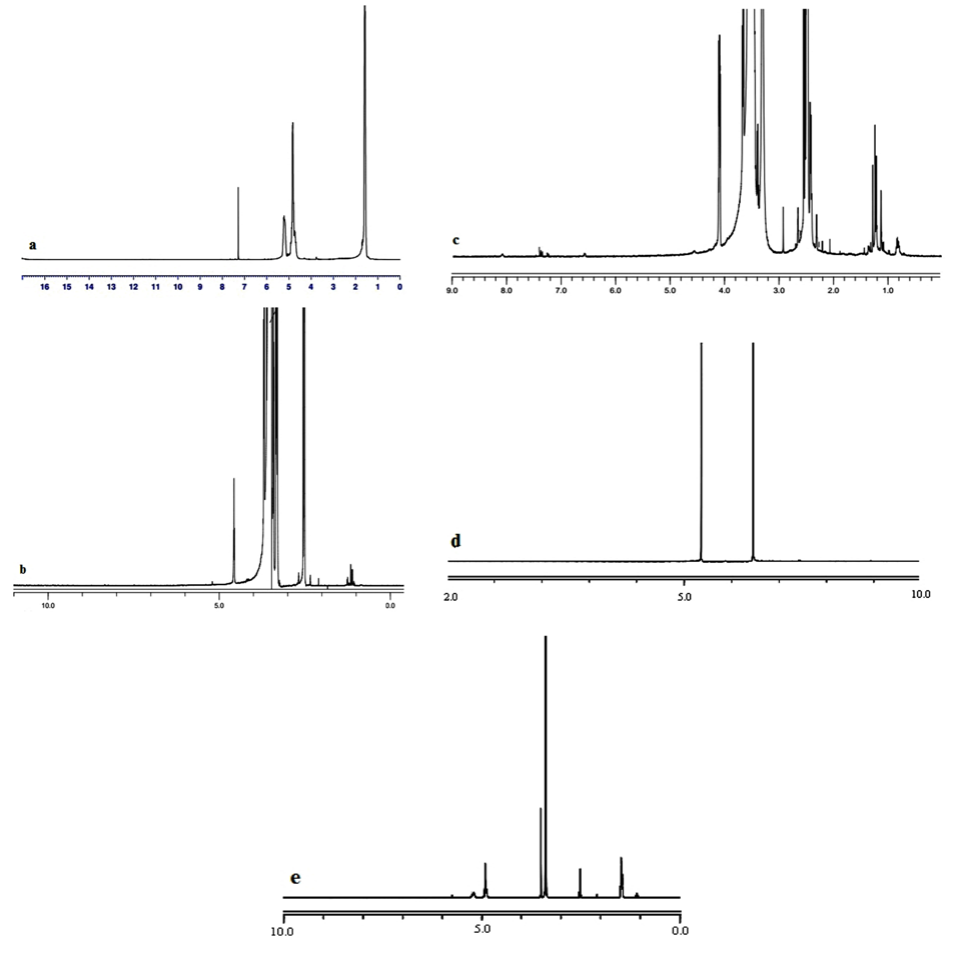
^1^H-NMR of the PLGA (a), PEG (b), PEG-Succinic anhydride (c), PEG-Succinic anhydride-GalN (d), and PEG- Succinic anhydride- GalN-PLGA (e).

The results of FTIR (Figure 1) and ^1^H-NMR (Figure 2) analysis confirmed the conjugation of PLGA to GalN. The FTIR spectrum of PEG demonstrated peaks in the range of 1280-1364 cm^-1^, 3488, and 2885,which were related to OH stretching vibration, methylene groups, and absorbance spectra of C–H bonds, respectively. The O–H stretching vibration band was observed at 3510 cm^-1^ in the infrared spectrum of PLGA. The spectrum of PLGA shows a clear bimodal peak at 2997 cm^-1^ and 2954 cm^-1^ reflecting the peak of the aliphatic CH_2_ group. Stretching vibrations at 1759 cm^-1^ were related to C=O stretching vibration. The C–O stretching vibrations were observed at 1261, 1168, and 1091 cm^-1^. The spectrum of the GalN shows a broad peak at about 3298 cm^-1^ region, which was identified as the stretching vibration of O–H. Peaks at 1000-1300 cm^-1^ were related to C–O, and C–N stretching vibration. In the PEG-succinic spectrum, the stretching vibration of the carbonyl group of succinic acid was observed at 1735 cm^-1^, which confirms the esterification reaction between PEG and succinic acid. The stretching vibration peak was observed at 1639 cm^-1^, that confirms the amidation reactions of conjugating GalN to succinic acid. The reaction between PEG succinic-GalN and PLGA was recognized by the presence of amide bands at 1631 cm^-1^ and ester bands at 1759 cm^-1^ in the final product spectrum.

The ^1^H-NMR spectrum of GalN showed a resonance at 4.79-5.29 ppm, which was assigned to the H1. The peak at 2.98 ppm was corresponded to H_2_. The peaks in the range of 3-4 ppm were characteristic of H3,H4, H5, and H6 protons. The resonances centered at 1.47, 4.92, and 5.19 ppm of the ^1^H-NMR spectrum of PLGA were related to the methyl group (CH_3_), the methylene group (CH_2_), and the CH group, respectively. In PEG spectrum, the methylene groups was observed at 3.54 ppm. The spectrum of the PEG-succinic acid show peaks at 3.51 and 3.68 ppm, which were identified as the methylene groups of PEG.Also, peak at 4.1 ppm was related to methylene group (CH_2_) attached to the ester group.Peak at 3.54 ppm was characteristic of methylene group of PEG, in PEG-succinic-GalN spectrum. As well as, the methylene group attached to the ester group displayed a peak at 4.1 ppm.Due to the formation of the amide band the high frequency shift was perceived for H_2_ of GalN. The final product showed the methylene group (CH_2_) of the glycolide acid, CH group of D, L-lactide monomer, and methyl group (C–H_3_) of D, L-lactide monomer at 4.92, 5.28, and 1.56 ppm, respectively. The overlapping between methylene groups of the PEG and H3,H4, H5, and H6 of GalN were appeared in the range of 3 to 4 ppm.

### Characteristic morphological of NPs

**Figure 3:**
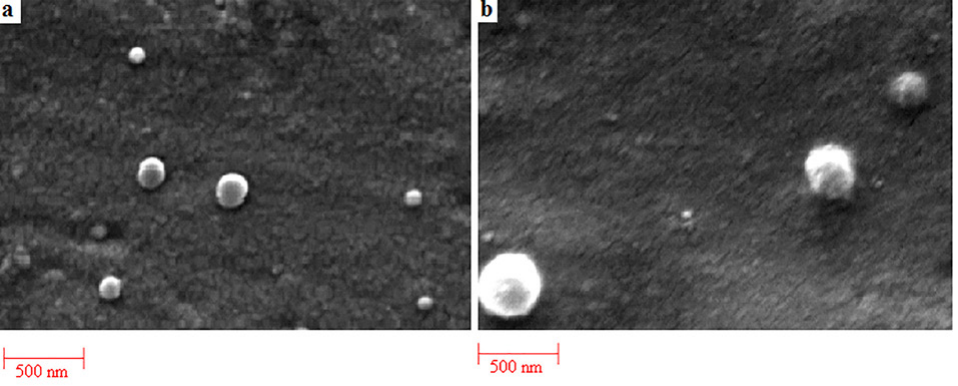
SEM image of Amphotericin B-loaded PLGA NPs (a) and Amphotericin B-loaded PLGA-GalN (b) NPs with the drug to polymer ratio of 1:6.

The physicochemical properties of NPs have been assessed in terms of size, polydispersity, surface charge and morphology. The size of amphotericin B loaded PLGA and PLGA-GalN NPs at various ratios is exhibited in Table 1. At higher polymer concentrations, larger particles were obtained. Similar results were obtained for naproxen-PLGA NPs.^24^ Conjugation of GalN into PLGA significantly enhanced the size of NPs, which can be due to the presence of GalN on the surface of NPs.^25^ There was no significant difference between the surface potential charge values of PLGA and PLGA-PEG NPs. SEM images (Figure 3) of the PLGA and PLGA-GalN NPs showed the spherical morphology with narrow particle size distribution.

### Nanoparticle drug loading

As shown in Table 1, raising the concentration of polymer leads to increment of the production yields and drug loading of NPs. This result may be due to the increasing viscosity of the internal organic phase and consequently decreasing the diffusion coefficient of the drug at higher polymer ratios. As well as, some of residual drug is lost on the NPs surface during the washing procedure when higher polymer ratios were applied.^26^ No obvious difference was detected between PLGA-GalN NPs and PLGA NPs for drug loading and NPs production yield (Table 1).

**Table 1:** Polydispersity (±SD), mean (±SD) particle diameter, zeta potential, encapsulation efficiency (±SD), and production yield of the various PLGA and PLGA-GalN NPs

**Formulations**	**Polydispersity**	**Mean particle size (nm)**	**Zeta potential (Mv)**	**Encapsulation efficiency (%)**	**Production yield (%)**
1:1 Drug:PLGA	0.21 ± 0.03	238.2±7.59	- 0.231	68.185% ± 1.9	67 %
1:3 Drug:PLGA	0.24 ± 0.014	174.46±3.49	- 0.285	75.69% ± 0.4	72 %
1:6 Drug:PLGA	0.13 ± 0.012	174.1± 4.188	0.365-	73.05 % ± 0.6	77 %
1:6 Drug:PLGA-GalN	0.286± 0.03	255.6± 4.0819	- 0.456	75.04 ± 0.95	71 %

### DSC results

**Figure 4:**
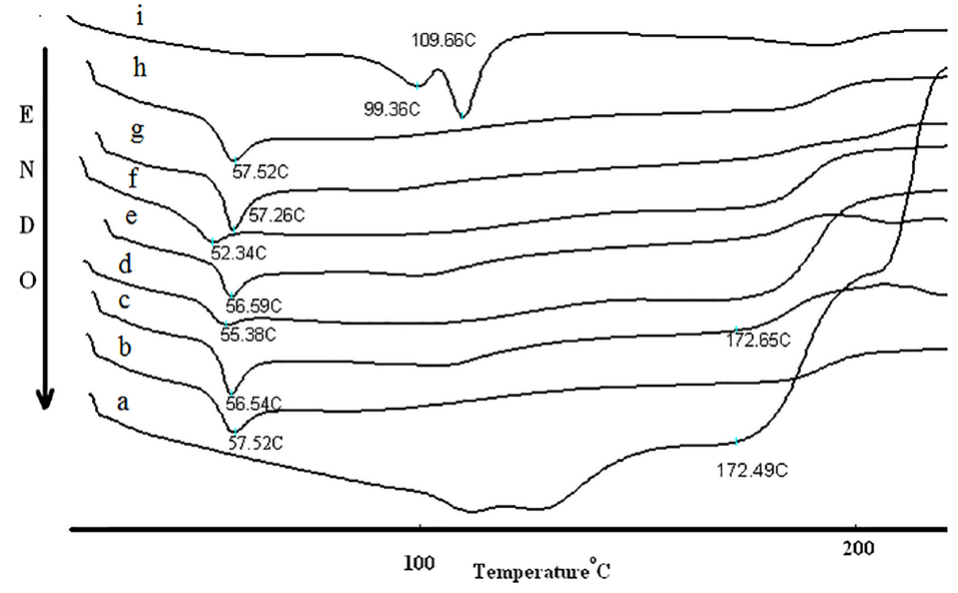
DSC curve of the intact Amphotericin B powder (a), PLGA(b), NPs of drug: PLGA 1:1 (c), physical mixture of drug: PLGA 1:1 (d), NPs of drug: PLGA 1:3 (e), physical mixture of drug: PLGA 1:3 (f), NPs of drug: PLGA 1:6 (g), physical mixture of drug: PLGA 1:6 (h), and NPs of drug: PLGA-GalN 1:6(i).

The DSC curve and melting temperature of amphotericin B, PLGA, NPs and their physical mixtures were illustrated in Figure 4. Amphotericin B thermogram revealed a peak at 172.98°C due to the melting of amphotericin B. The reduction of melting point of amphotericin B occurs in all physical mixtures because of the colligative properties. The decrease in enthalpy of fusion in all NPs and physical mixture compare with intact amphotericin B showed that the crystalline drug was transformed to an amorphous. DSC results (Figure 4) showed a weak endothermic enthalpy for NPs when compared with physical mixture and intact amphotericin B. The melting enthalpy of drug was also decreased by increasing amount of polymer. These results can be due to the solubility of amphotericin B in the molten polymers and the dilution of amphotericin B. The significant decrease in the melting enthalpy of fusion in all NPs compare with physical mixture indicates the presence of intermolecular hydrogen bands and interaction between drug and polymers. The results showed that the enhanced PLGA-GalN NPs melting point was related to enhancement of the functional groups and hydrogen bonding reinforcing effect.

### X*-*ray powder diffractions 

**Figure 5:**
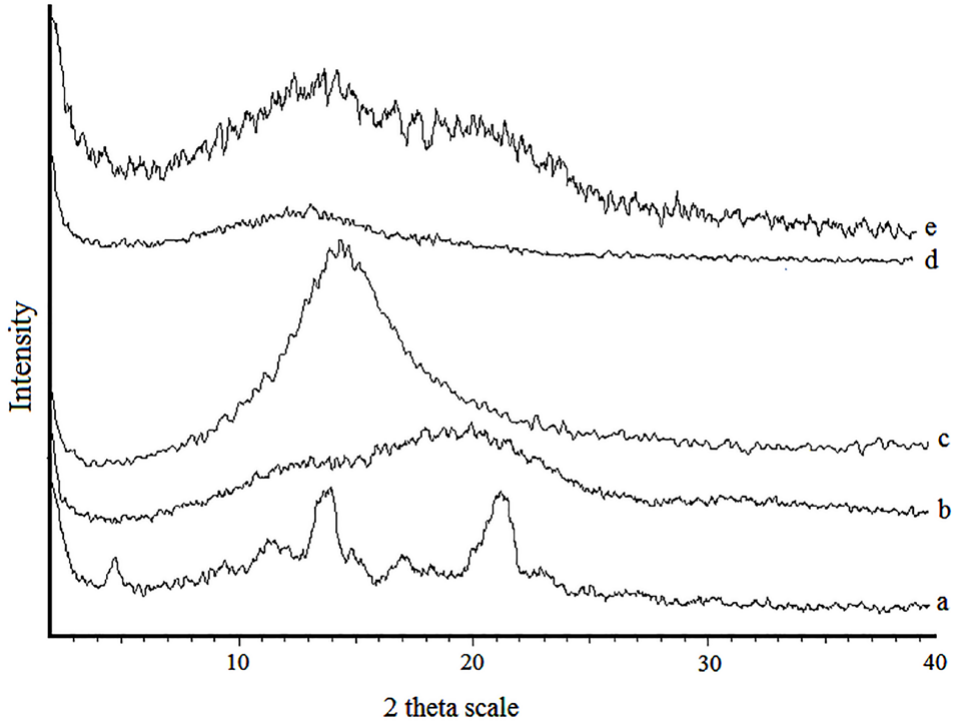
Powder X-ray diffraction of intact Amphotericin B powder (a), PLGA (b), Amphotericin B-loaded NPs with the drug PLGA ratio of 1:3 (c), 1:6 (d), and Amphotericin B-loaded NPs with the drug PLGA-GalN ratio of 1:6 (e).

The X-ray diffraction patterns are shown in Figure 5. The XRD patterns of intact amphotericin B revealed several distinct diffraction peaks in the 2θ°at 17.38, 21.4 and 25 that are indicative of their crystalline character.^27^ No major peak was observed for PLGA because the amorphous nature of PLGA.^28^ Absence of crystalline peaks of amphotericin B in the diffraction pattern of PLGA and PLGA-GalN NPs confirmed amorphous state. It can be seen that increasing the amount of polymer makes NPs more amorphous. These results are in agreement with DSC study.

### In vitro release study

**Figure 6:**
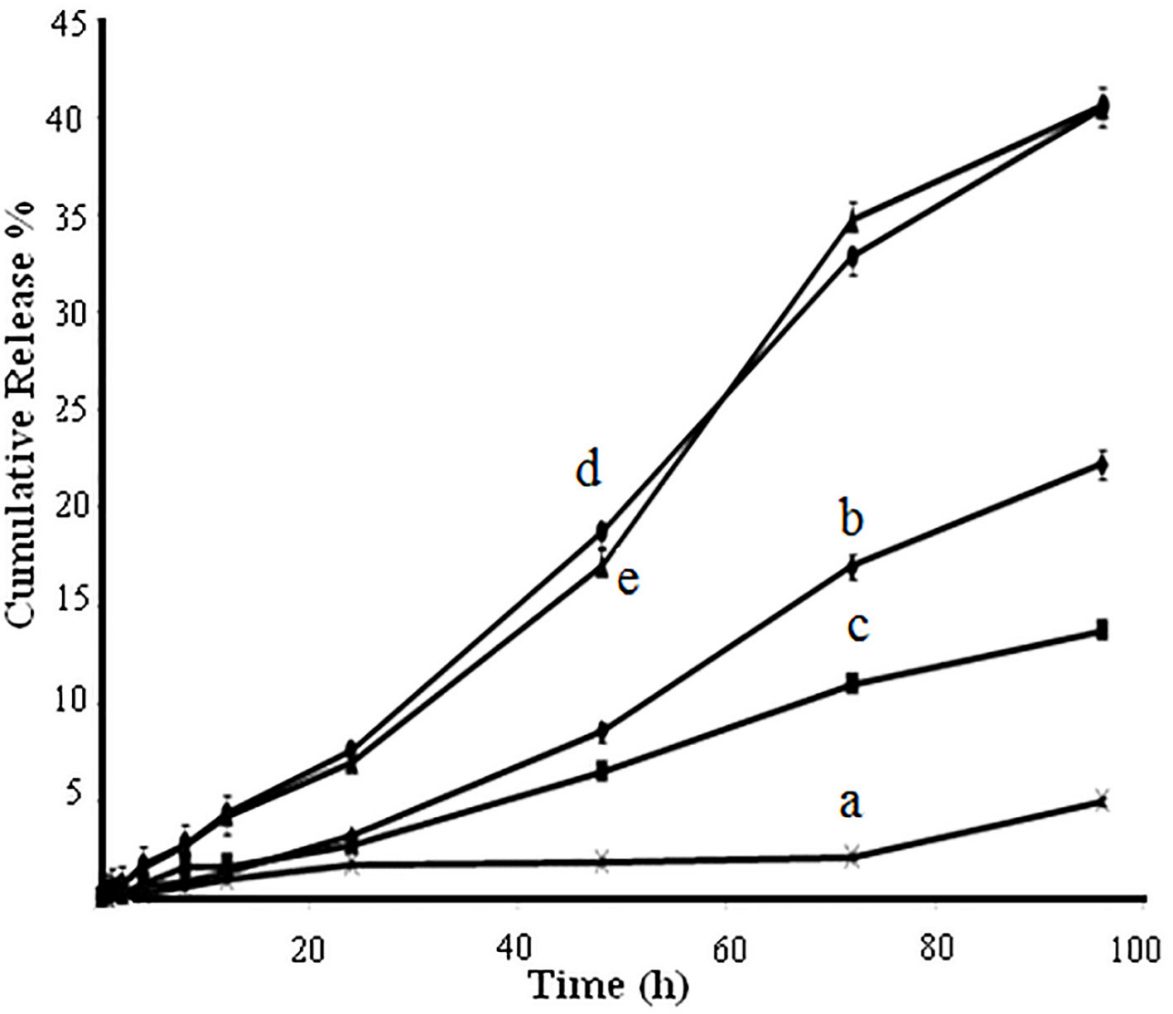
Dissolution profiles of intact Amphotericin B powder (a) and Amphotericin B-loaded NPs with drug to PLGA ratio of 1:1 (b), 1:3 (c), 1:6 (d), and drug to PLGA-GalN ratio of 1:6b (e).

Figure 6 shows the profiles of in vitro and in vivo data for intact amphotericin B powder and the formulated amphotericin B loaded polymeric NPs. In vitro release of NPs demonstrated a slower drug release profile than the intact drug. The release rate of amphotericin B was observed for intact powder, PLGA NPs with drug to polymer ratios of 1:1, 1:3, 1:6, and PLGA-GalN NPs with drug to polymer ratio of 1:6. The cumulative release of amphotericin B was 67%, 72%, 77%, and 71% from PLGA NPs with drug to polymer ratios of 1:1, 1:3,1:6, and PLGA-GalN NPs with drug to polymer ratio of 1:6, respectively. The gradual release rate of amphotericin B from all NPs which is dependent on polymer concentration was observed over the 96 h of study. The results show that the amphotericin B release rate increase with increasing the polymer concentration. This enhancement of drug release rate by increasing the amount of polymer could be due to an increase in crystallinity of NPs. These results are consistent with the results presented by Nahar and Jain.^29^ No significant difference was found between release profiles of PLGA and PLGA-GalN NPs. However, the presence of the hydrophilic functional groups has significantly increased the release rate. An increase in the size of NPs led the drug release to decrease.^30^

Mathematical models to predict drug release were obtained by fitting the drug release data to various release kinetic models. Among measured mathematical models, Zero order correlation was the best fitted model for PLGA NPs with the ratio of 1:3 drug to polymer. The release profiles were observed followed by Papias model for PLGA NPs with drug to polymer ratio of 1:1 and Weibull model for NPs with drug to polymer ratio of 1:6.

### Antifungal activity

PLGA and PLGA-GalN NPs without any drug had no detectable antifungal effect. The evaluation of MIC showed that the lowest MIC was recorded about 3 µg/mL for PLGA-PEG-GalN NPs with polymer/drug ratio 1:6. MICs of PLGA NPs at drug/polymer ratio 1:6 was equal to 4 μg/mL. The MIC of drug loaded PLGA NPs was two-fold less in comparison with the free drug (MIC = 8). These results indicated that the prepared PLGA-PEG-GalN NPs were more potent against *C. albicans* (MIC=3). PLGA-PEG-GalN NPs showed higher antifungal efficacy than pure amphotericin B and PLGA NPs (MIC=4). These results can be attributed to increased adhesion of PLGA-GalN NPs on the fungal cells which consequently increased drug amount in the membrane of the fungal cells.

Amphotericin B NPs tested for antifungal activity showed better results than the pure drug solution. According to Figure 6, these results could be due to increased solubility of drug-loaded NPs.

## Conclusion

In summary, GalN conjugated PLGA NPs were successfully synthesized. Amphotericin B loaded PLGA and PLGA-GalN NPs with various drug/polymer ratio was prepared by quasi emulsion solvent diffusion method. FT-IR and ^1^H-NMR spectra confirmed the conjugation of PLGA to GalN. Transition metal-carbonyls group frequencies and secondary amine N–H bend ensured GalN modification on PLGA NPs. According to the results, GalN conjugated PLGA polymer could be used as useful amphotericin B carriers. The particle size of PLGA-GalN NPswereless than 250 nm (100-250 nm), depending on the drug-polymer ratio. The size of NPs can be enhanced by increasing the polymer concentrations.

The absence of crystalline peaks of amphotericin B in the X-ray diffraction patterns of PLGA and PLGA-GalN NPs and decrease in enthalpy of fusion in DSC thermograms of NPs confirmed the amorphous state. All NPs showed potent antifungal activity and PLGA-GalN NPs exhibited good antifungal activity greater than PLGA NPs. These results indicate that PLGA-GalN NPs have proper antifungal potency. This system can be used to provide targeted delivery for amphotericin B to improve its antifungal activity.

## Ethical Issues

Not applicable.

## Conflict of Interest

None.

## Acknowledgments

The authors gratefully acknowledge the financial support of this study by the Kermanshah University of Medical Sciences, Kermansha, Iran
